# 6q deletion is frequent but unrelated to patient prognosis in breast cancer

**DOI:** 10.1007/s12282-021-01301-5

**Published:** 2021-10-08

**Authors:** Patrick Lebok, Hannah Bönte, Martina Kluth, Christina Möller-Koop, Isabell Witzel, Linn Wölber, Peter Paluchowski, Christian Wilke, Uwe Heilenkötter, Volkmar Müller, Barbara Schmalfeldt, Ronald Simon, Guido Sauter, Luigi Terracciano, Rainer Horst Krech, Albert von der Assen, Eike Burandt

**Affiliations:** 1 Institute of Pathology, University Medical Center Hamburg-Eppendorf, Martinistr. 52, 20246 Hamburg, Germany; 2 Department of Gynecology, University Medical Center Hamburg-Eppendorf, Hamburg, Germany; 3 Department of Gynecology, Regio Clinic Pinneberg, Pinneberg, Germany; 4 Department of Gynecology, Regio Clinic Elmshorn, Elmshorn, Germany; 5 Department of Gynecology, Clinical Centre Itzehoe, Itzehoe, Germany; 6 Department of Pathology, Basel University Clinics, Basel, Switzerland; 7 Institute of Pathology, Clinical Centre Osnabrück, Osnabrück, Germany; 8 Breast Centre Osnabrück, Osnabrück, Germany

**Keywords:** Breast cancer, 6q15 deletion, TMA, Prognosis, Genomic alterations

## Abstract

**Background:**

Deletions involving the long arm of chromosome 6 have been reported to occur in breast cancer, but little is known about the clinical relevance of this alteration.

**Methods:**

We made use of a pre-existing tissue microarray with 2197 breast cancers and employed a 6q15/centromere 6 dual-labeling probe for fluorescence in situ (FISH) analysis

**Results:**

Heterozygous 6q15 deletions were found in 202 (18%) of 1099 interpretable cancers, including 19% of 804 cancers of no special type (NST), 3% of 29 lobular cancers, 7% of 41 cribriform cancers, and 28% of 18 cancers with papillary features. Homozygous deletions were not detected. In the largest subset of NST tumors, 6q15 deletions were significantly linked to advanced tumor stage and high grade (*p* < 0.0001 each). 6q deletions were also associated with estrogen receptor negativity (*p* = 0.0182), high Ki67 proliferation index (*p* < 0.0001), amplifications of HER2 (*p* = 0.0159), CCND1 (*p* = 0.0069), and cMYC (*p* = 0.0411), as well as deletions of PTEN (*p* = 0.0003), 8p21 (*p* < 0.0001), and 9p21 (*p* = 0.0179). However, 6q15 deletion was unrelated to patient survival in all cancers, in NST cancers, or in subsets of cancers defined by the presence or absence of lymph-node metastases.

**Conclusion:**

Our data demonstrate that 6q deletion is a frequent event in breast cancer that is statistically linked to unfavorable tumor phenotype and features of genomic instability. The absence of any prognostic impact argues against a clinical applicability of 6q15 deletion testing in breast cancer patients.

## Introduction

Breast cancer is the most common malignancy detected in women [1]. Surgical removal of the cancer represents the standard of care. Whether or not adjuvant systemic therapy is given depends on the perceived aggressiveness of the removed cancer. Currently established prognostic parameters mainly include histological grade, tumor size, presence of lymph-node metastasis, tumor cell proliferation (Ki67-labeling index; Ki67 LI), as well as hormonal receptor and HER2 status [2]. Additional molecular parameters are analyzed in many patients [3–5]. Commercial molecular classifiers are based on multiplexed analyses of the RNAs of 21–70 gene products [6–8]. These purely RNA-based tests share the disadvantage that gradual changes of each parameter must be measured, and that these measurements are strongly dependent on tumor cell purity. As next-generation sequencing (NGS) is getting less expensive, it is expected that alternative and potentially better prognostic tests will be increasingly based on DNA analyses including a global assessment of structural rearrangements and gene mutations. NGS tests can analyze biomarkers with yes/no answers such as presence or absence of individual mutations or deletions. In other tumor types, especially in prostate cancer—another important hormone dependent cancer—various chromosomal deletions have been shown to have substantial prognostic relevance [9–12]. One of these is deletion of 6q12-q21, which is also commonly found in breast cancer. Studies using classical comparative genomic hybridization in 16–34 patients [13, 14], array-based copy-number screening assays in 28 patients [15], or loss of heterozygosity (LOH) analysis 42–83 patients [14, 16–18] reported 6q deletions in 6–50% of breast cancers. Some of these studies have described an association of 6q deletions with unfavorable tumor phenotype [14, 19].

To better understand the clinical relevance of 6q deletions in breast cancer, we utilized a pre-existing breast cancer tissue microarray (TMA) containing more than 2000 cancers. Our data show that 6q deletion is frequent but unrelated to patient prognosis in breast cancer.

## Materials and methods

### Breast cancer tissue microarray (TMA)

A pre-existing tissue microarray (TMA) was used for this study [20]. The TMA contained 2197 human breast cancer tissue punches (diameter 0.6 mm) from paraffin-embedded tissue specimens fixed in 4% neutral buffered formalin. The donor blocks used for TMA construction were collected from the archives of the Institute of Pathology of the University Hospital Basel, the Institute for Clinical Pathology in Basel, and the Triemli Hospital in Zurich. Tumors were collected consecutively, and all slides from the tumors were reviewed by specialized pathologists to define the histologic grade according to Elston and Ellis [21] and the histologic tumor type. The use of the specimens and data for research purposes were approved by the Ethics Committee of the Basel University Hospital. Survival data were either obtained from the cancer registry of Basel or collected from the patients attending physicians. The median patient’s age was 63 (range 26–101) years. Raw survival data were available from 1982 patients (713 patients with and 1508 without event). The mean follow-up time was 63 months (range 1–176 months). Tumor size and nodal status were obtained from the primary pathology reports. Four micrometer sections of the TMA blocks were transferred to an adhesive-coated slide system (Instrumedics Inc., Hackensack, New Jersey) for FISH analysis. Molecular data used in this study were available from previously published studies. These included data obtained by FISH for amplification of HER2 [20, 22]*,* CCND1 [20], MDM2 [20], and cMYC [20, 22] as well as for deletions of PTEN [23], 8p21 [24], and 9p21 [25] and data obtained by IHC for estrogen receptor (ER) and progesterone receptor (PR) expression as well as Ki67-labeling index (Ki67 LI) [20, 26]. Molecular subtypes (Luminal A, B, HER2, basal cell type) were defined according to the St. Gallen (2011) criteria [27].

### Fluorescence in situ hybridization

**Fig. 1:**
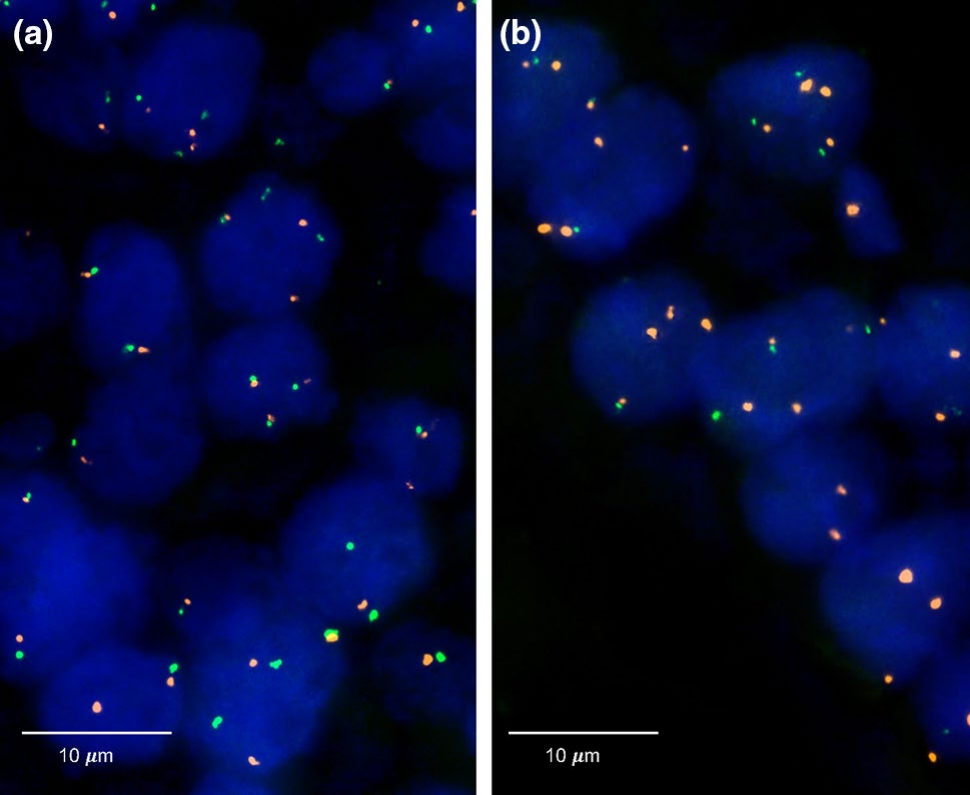
Representative images of FISH findings using the 6q15 deletion probe. **a** Normal 6q15 copy numbers as indicated by two green 6q15 signals and two orange centromeres 6 signals and **b** heterozygous deletion as indicated by the lack of one green 6q15 signal and two orange centromere 6 signals

Four micrometer TMA sections were used for FISH. For proteolytic slide pretreatment, a commercial kit was used (paraffin pretreatment reagent kit; Abbott, Wiesbaden, Germany). TMA sections were deparaffinized, air-dried, and dehydrated in 70%, 85%, and 100% ethanol, followed by denaturation for 5 min at 74 °C in 70% formamide 2 × SSC solution. The FISH probe set consisted of a spectrum-green labeled 6q15 (MAP3K7) probe (made from a mixture of BAC RP3-470J08 and BAC RP11-501P02), and a spectrum-orange labeled commercial centromere 6 probe (#06J36-06; Abbott, Wiesbaden, Germany) as a reference. Hybridization was performed overnight at 37 °C in a humidified chamber. Slides were subsequently washed and counterstained with 0.2 µmol/L 4’-6-diamidino-2-phenylindole in antifade solution. Stained slides were manually interpreted with an epifluorescence microscope, and the predominant FISH signal numbers were recorded in each tissue spot. The presence of fewer 6q15 signals than centromere 6 probe signals in at least 60% tumor nuclei was considered a heterozygous deletion. These thresholds were based on our previous study analyzing *PTEN* deletions on a prostate cancer TMA where our approach resulted in a 100% concordance with array comparative genomic hybridization (CGH) data [12]. Complete absence of 6q15 signals in all tumor cells, but presence of centromere 6 and 6q15 signals in adjacent normal cells, was considered a homozygous deletion. Tissue spots lacking any detectable 6q15 signals in all cells (tumor and normal cells or tumor cells only but no normal cells present) were excluded from analysis because of a lack of an internal control for successful hybridization of the 6q15 probe. Representative images of 6q15 FISH results are shown in Fig. 1.

### Statistics

Statistical calculations were performed with JMP 14 software (SAS Institute Inc., NC, USA). Contingency table analysis and Chi-square test were used to study the relationship between FISH results and clinicopathological variables. Kaplan–Meier plots were used to estimate overall survival and the statistical significance was determined by the log-rank test. The log-rank test was applied to test the significance of differences between stratified survival functions.

## Results

### Technical issues

A total of 1099 (50%) of arrayed cancer samples were analyzable by FISH. Reasons for non-informative results included non-interpretable FISH signals (589, 26%), lack of tumor cells in the tissue spot (224, 10%), or absence of tissue spot on the TMA section (309, 14%).

### 6q15 deletion and breast cancer phenotype

Heterozygous 6q15 deletions were found in 202 (18%) interpretable breast cancers. Representative images from cancers with and without 6q15 deletions are given in Fig. 1a,b. Homozygous 6q15 deletion was not observed. 6q15 deletions were found in 19% of 804 NST cancers, 3% of 29 tubular cancers (*p* = 0.0076 vs NST), 7% of 41 cribriform cancers (*p* = 0.0338 vs NST), and 28% of 18 cancers with papillary features (*p* = 0.5508 vs NST). If all cancers were jointly analyzed, deletion of 6q15 was significantly linked to advance tumor stage (*p* = 0.0315) and high histopathological grade (*p* < 0.0001). These associations also held true for BRE grade in the largest subset of NST cancers (*p* < 0.0001). In addition, 6q15 deletions were significantly linked to the subset of estrogen receptor (ER) negative breast cancers: deletion was found in 24% of ER negative but only in 17% of ER-positive breast cancers (*p* = 0.0182) and to the molecular subtypes of HER2-positive and basal cell type cancers (*p* = 0.0062). 6q15 deletion was unrelated to the presence of lymph-node metastases and progesterone receptor status. All results are summarized in Table 1.

**Table 1:** Relationship between 6q15 deletion and histopathological parameters in breast cancer

		Analyzable (n)	6q15 FISH result		*p* value
			Normal (%)	Deletion (%)	
All cancers		1099	82%	18%	
Histology	No special type	804	81%	19%	
	Lobular carcinoma	104	87%	13%	
	Cribriform carcinoma	41	93%	7%	**0.0338
	Medullary carcinoma	33	82%	18%	
	Tubular carcinoma	29	97%	3%	**0.0076
	Papillary carcinoma	18	72%	28%	**0.5508
	Mucinous carcinoma	32	75%	25%	
	Other rare types*	56	73%	27%	
pT stage	pT1	351	84%	16%	0.0315
	pT2	552	82%	18%	***0.0756
	pT3	55	69%	31%	
	pT4	134	77%	23%	
BRE grade	Grade 1	266	89%	11%	< 0.0001
	Grade 2	373	82%	18%	***0.0007
	Grade 3	379	74%	26%	
Nodal stage	pN0	460	81%	19%	0.2561
	pN1	391	83%	17%	***0.1528
	pN2	69	74%	26%	
	pN3	920	81%	19%	
ER status	Negative	263	76%	24%	0.0182
	Positive	804	83%	17%	***0.0897
PR status	Negative	663	80%	20%	0.2071
	Positive	365	84%	16%	***0.5111
Molecular subype	Luminal A	105	91%	9%	0.0062
	Luminal B	660	83%	17%	***0.0619
	HER2	116	77%	23%	
	Basal cell type	240	78%	22%	

*Including adenoid-cystic carcinoma, apocrine carcinoma, atyp medullary carcinoma, carcinosarcoma, clear cell carcinoma, histiocytic carcinoma, lipid-rich carcinoma, lipid-rich or histiocytic carcinoma, metaplastic carcinoma, neuroendocrine carcinoma, signet ring carcinoma, and small cell carcinoma. **vs. cancers of no special type, ***only in the subset of NST cancers

### 6q15 deletion and tumor cell proliferation

Data on tumor cell proliferation, as determined by immunohistochemical analysis of the Ki67 antigen, were available from a previous study using the same TMA [20]. Deletion of 6q15 was tightly linked to a high Ki67 LI if all cancers were jointly analyzed (*p* < 0.0001). This association was not independent of histological grade. All results are summarized in Table 2.

**Table 2:** Relationship between 6q15 deletion and tumor cell proliferation (Ki67-labeling index) in all cancers and the subset of cancers with identical histological grade

		Analyzable (n)	Ki67LI		*p* value
			Mean	Std. deviation	
All cancers	6q15 normal	798	28.56	0.52	< 0.0001
	6q15 deletion	177	33.32	1.10	
Grade 1	6q15 normal	205	18.81	0.70	0.0432
	6q15 deletion	23	23.26	2.08	
Grade 2	6q15 normal	275	26.34	0.68	0.1027
	6q15 deletion	64	28.89	1.41	
Grade 3	6q15 normal	251	39.05	0.94	0.8346
	6q15 deletion	84	39.44	1.63	

### Prognostic significance of 6q15 deletion

**Fig. 2:**
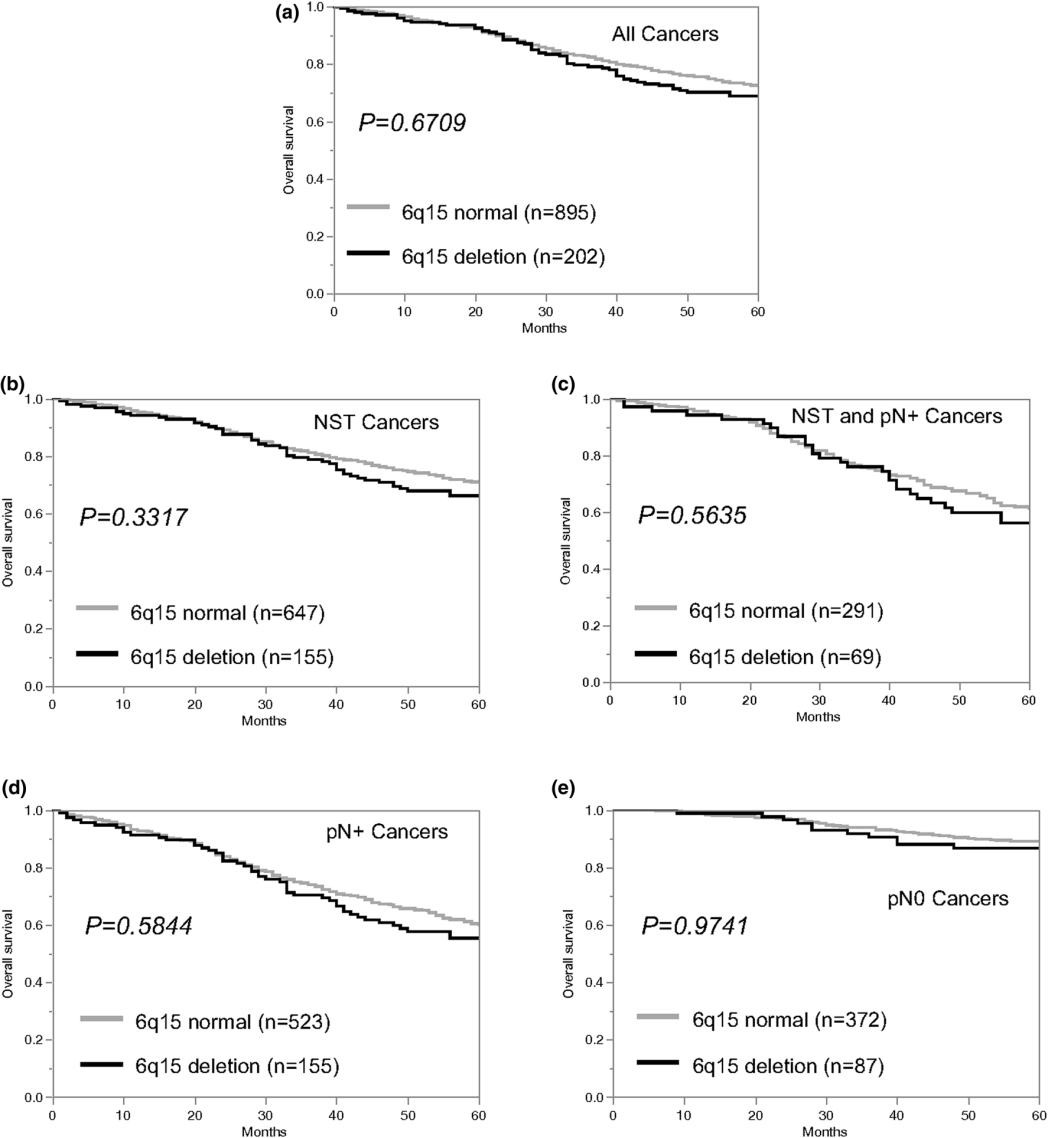
Relationship between 6q15 deletion and overall survival in **a** all cancers, **b** no special type (NST) cancers, **c** NST and nodal positive cancers, **d** nodal positive cancers, and **e** nodal negative cancers

Data on raw survival were available from 1097 cancers with interpretable 6q15 FISH results. The presence of 6q15 deletion was largely unrelated to shortened overall survival if all cancers were jointly analyzed (*p* = 0.6709, Fig. 2a), as well as in the subsets of cancers of No Special Type (NST, *p* = 0.3317, Fig. 2b), in the subset of NST cancers with nodal metastases (*p* = 0.5635, Fig. 2c), and in the subset of cancers with or without nodal metastases (*p* = 0.5844 for pN positive and *p* = 0.9741 for pN negative; Fig. 2d-e).

### 6q15 deletion and other genomic alterations in breast cancer

**Fig. 3:**
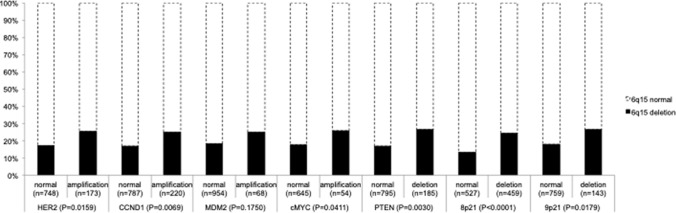
Relationship between 6q15 deletion and key genomic alterations in breast cancer

HER2, CCND1, MYC, and MDM2 amplification, as well as PTEN, 8p21, and 9p21 deletion results were available from our previous studies. In total, FISH results on both 6q15 deletions and alterations of HER2, CCND1, MYC, MDM2, PTEN, 8p21, and 9p21 were available in subsets of 921 (HER2), 1007 (CCND1), 699 (MYC), 1022 (MDM2), 980 (PTEN), 986 (8p21), and 902 (9p21) cancers. Deletions of 6q15 were significantly linked to most of all alterations (*p* ≤ 0.04). For example, 6q15 deletion was found in 25% of 173 HER2-amplified cancers but only in 17% of 748 cancers with normal HER2 copy-number status (*p* = 0.0159), as well as in 26% of 185 PTEN deleted cancers but only in 17% of 795 cancers with normal PTEN status (*p* = 0.0030). No significant association was found between 6q15 deletion and MDM2 amplification (*p* = 0.1750). All results are summarized in Fig. 3.

## Discussion

The analysis of more than 1000 breast cancers using an FISH probe directed against a DNA sequence at 6q15 identified a heterozygous deletion in 18% of tumors. This finding is consistent with data from the ICGC/TCGA database describing 6q15 deletion in 26% of 2051 sequenced breast cancers from the METABRIC cohort [28]. Earlier published studies had reported 6q deletion frequencies between 6 and 50% in cohorts of 16–83 patients [13–19] and in 8 of 10 breast cancer cell lines [29]. A variety of methods had been applied in these studies including classical and array CGH, LOH analysis, and FISH. We are confident that our findings reflect the true frequency of 6q15 deletion in breast cancer, because fluorescence in situ hybridization (FISH) represents the gold standard for gene copy-number analysis. FISH is independent of the purity of cancer tissue and chromosomal aberrations such as polysomy. Deletions can be analyzed on a cell-by-cell basis, and abnormalities can be detected in a few cells or even a single cell. In our study, 6q15 deletion was defined as “fewer 6q15 signals than centromere 6 signals in at least 60% of all tumor cells”. These stringent criteria resulted in a 100% concordance of results found by FISH and comparative genomic hybridization in a previous PTEN study of our group in prostate cancer [12]. Our cut-off of 60% deleted cells is also supported by the observation that virtually all deleted cases had fewer gene signals than centromere signals in > 80% of cells and undeleted cases had usually fewer than 10% cells with fewer 6q15 signals than centromere signals.

The comparison of 6q15 deletions with histopathological tumor features revealed statistically significant associations. This was true for aggressive molecular subtypes including the HER2 and basal cell type and especially for the BRE grade. This is not surprising as a high BRE grade is characterized by a particular high degree of nuclear atypia, which—in turn—is often related to a high frequency of genomic alterations [30–32]. Accordingly, the rate of 6q15 deletions was particularly low in cancer subtypes characterized by low nuclear atypia such as tubular or lobular carcinoma and particularly high in medullary carcinoma, a tumor characterized by substantial nuclear atypia. The assumption that 6q15 deletions accumulate in tumors with an increased level of genomic instability which fits well with the significant associations found between 6q15 deletions and all other previously analyzed genomic aberrations such as amplifications of MYC; HER2, MDM2, and CCND1, as well as deletions of 8p, 9p21 and PTEN [20, 22–25]. The relationship of 6q15 deletions with all these aberrations was highly similar. All genomic changes occurred between 1.4 and 1.8 more often in 6q15 deleted than in 6q15 undeleted carcinomas. It appears thus more likely that these associations are caused by a general phenomenon such as “genomic instability” than by specific interactions between associated pathways. In an earlier study analyzing gene amplifications, we had already found that tumors carrying one amplification are significantly more prone to develop additional amplifications [33].

The 6q gene(s) driving cancer progression through inactivation has not been clearly identified. Copy-number data derived from the ICGC/TCGA database (www.cbioportal.org) [34] do not suggest a clear-cut minimal commonly deleted region in breast cancer, although highest frequencies (26%) are found in the interval between 85 Mb (6q14) and 100 Mb (6q21). An FISH probe for MAP3K7 had been selected for this study because of its location in the center of the 6q15 deletion and the known tumor suppressive function of MAP3K7 [35]. Other 6q15 genes with potential tumor suppressive functions for example include EEF1A1 [36], ZNF292 [37], SNORD50A [38], PRDM1 [39], CCNC [40], FOXO3 [41], WISP3 [42], and FRK [43]. It is of note, however, that inactivation of the second allele by homozygous deletion or inactivating mutation is virtually not existent. In the METABRIC [28] dataset, EEF1A1 or FOXO3 were the only genes for which homozygous deletions could be seen in 1.6% of more than 1,000 tumors. A classical tumor suppressive role of FOXO3 in a very small subset of breast cancers is further supported by recurrent mutations (*n* = 38, 1.4%), almost half of them being associated with deletions of the second allele. For all other 6q15 candidate genes, neither homozygous deletions nor recurrent mutations were described. 6q15 deletions—as other large genomic deletions—may thus exert their tumor promoting role through a reduced function of multiple genes within the deletion. Of note, the complete absence of large homozygous MAP3K7 deletions argues for one or several essential genes in the 6q15 area for which complete inactivated is not consistent with cell survival.

Irrespective of which gene(s) are affected by 6q15 deletions, our data do not suggest a substantial impact of a dysfunction of these for the clinical course of affected patients. The complete lack of differences in patient outcome between 6q15 deleted and undeleted cancers would even be consistent with 6q15 deletions representing an irrelevant “passenger” lesion” in breast cancer. However, given the prominent role of 6q15 deletions in various other cancer types, including a clear-cut prognostic impact in prostate cancer [44, 45], we would not anticipate this deletion to be meaningless. There are examples of critical molecular events for cancer development, such as TMPRSS2–ERG fusions, the most frequent molecular alteration in prostate cancer occurring in about 50% of cases, which are completely unrelated to disease outcome [46]. Our data on “only” 1099 successfully analyzed cancers do not exclude a clinically relevant role of 6q15 alterations in a morphologically, molecularly, or clinically (treatment) defined subgroup of cancers.

In conclusion, these data identify 6q15 deletions as a frequent event in breast cancer. Despite statistically significant associations with important histological and molecular features, 6q15 deletions are largely unrelated to patient outcome. 6q15 deletion analysis does not appear to have potential clinical utility.
